# Supplementary figures and images for: Molecular phylogeny and cryptic morphology: A combined approach to taxonomic novelties in Polycarpaea (Caryophyllaceae) from Vietnam
Source: PLoS One. 2024 Oct 16;19(10):e0301407. doi: 10.1371/journal.pone.0301407 (PMC11482727; doi:10.1371/journal.pone.0301407)

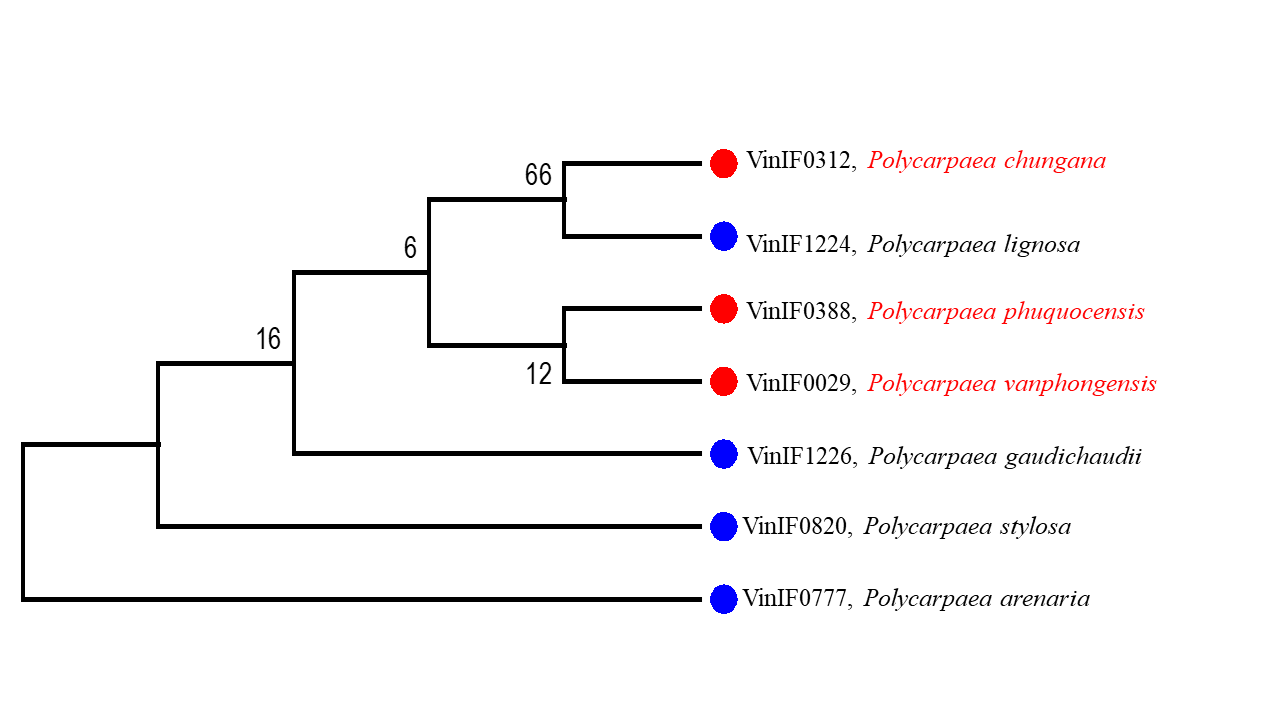

Supplement: S1 Fig — (TIF) [file pone.0301407.s001.tif]

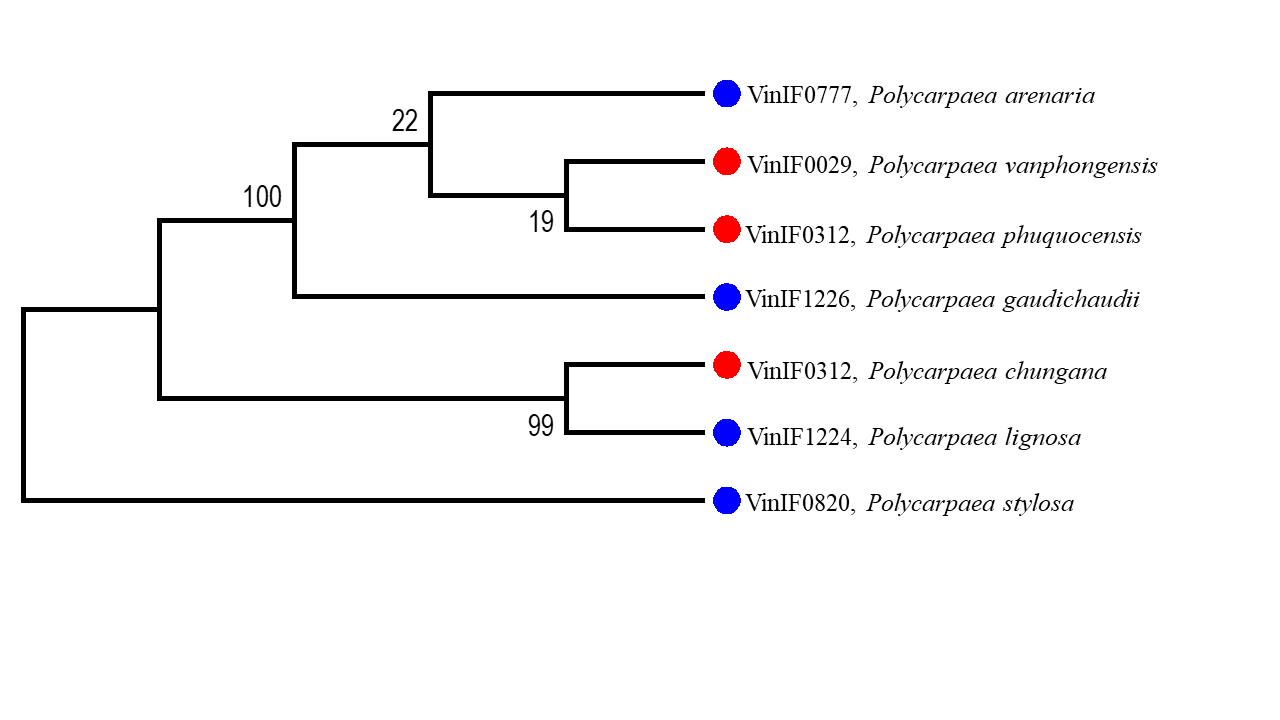

Supplement: S2 Fig — (TIF) [file pone.0301407.s002.tif]

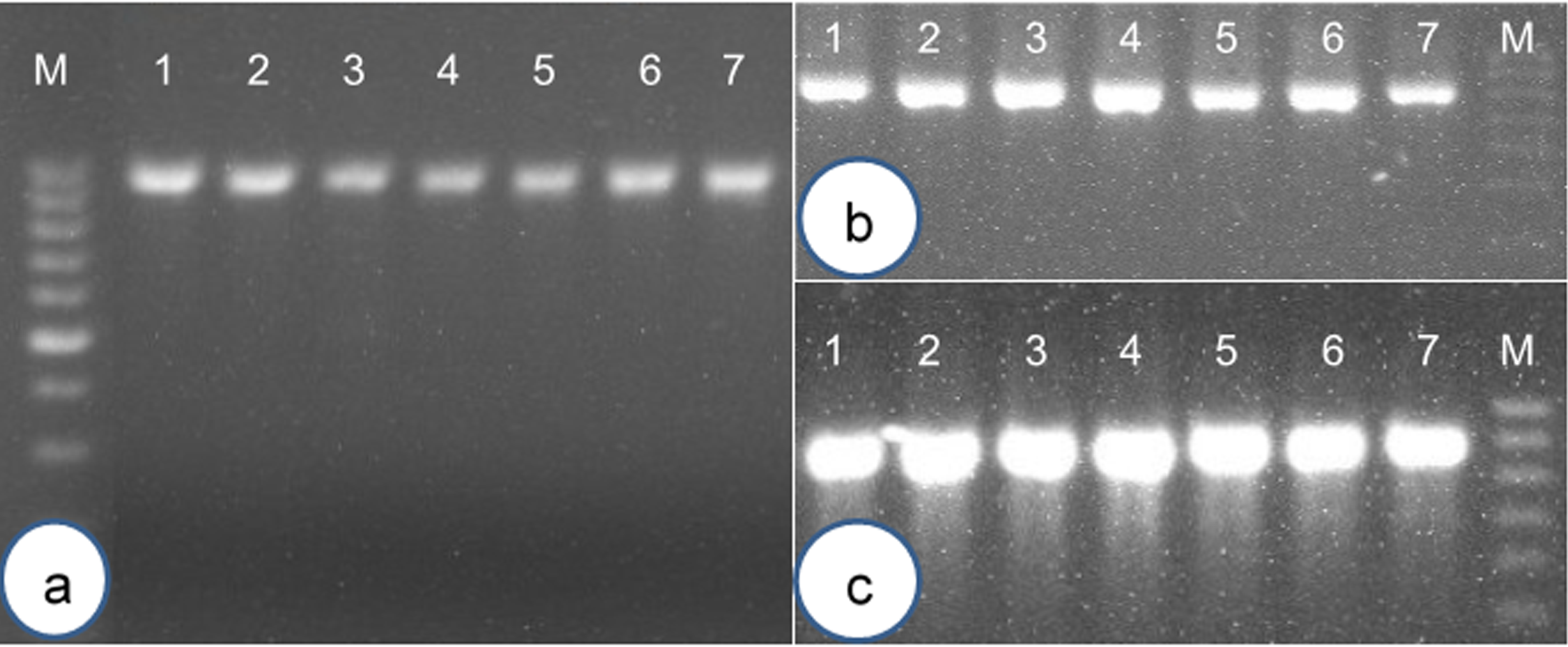

Supplement: S3 Fig — a. total extracted genomic DNA. b. ITS1-5.8S rRNA-ITS2 PCR products. c. rps16 intron PCR products. M. DNA ladder (Bioline a. 1kb. b. c. 100bp). (TIF) [file pone.0301407.s003.tif]

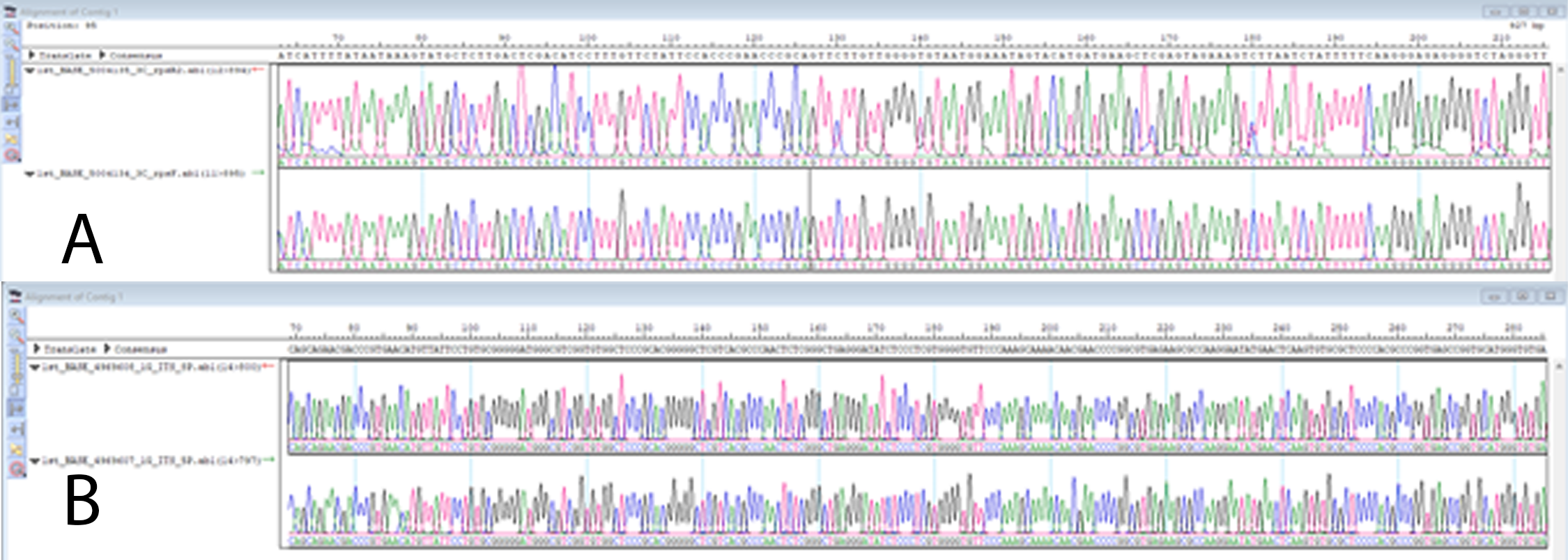

Supplement: S4 Fig — A, ITS1-5.8S rRNA-ITS2 sequences; B, rps16 intron sequences. (TIF) [file pone.0301407.s004.tif]
