# Supplementary material for: Molecular phylogeny and cryptic morphology: A combined approach to taxonomic novelties in Polycarpaea (Caryophyllaceae) from Vietnam
Source: PLoS One. 2024 Oct 16;19(10):e0301407. doi: 10.1371/journal.pone.0301407 (PMC11482727; doi:10.1371/journal.pone.0301407)
Supplement: S1 Table — (DOCX) [file pone.0301407.s005.docx]

**S1 Table.** The quality and quantity of total extracted genomic DNA

| **Sample No.** | **Species** | **DNA concentration (ng/µL)** | **Ratio**  **260/280** |
| --- | --- | --- | --- |
| 1 | *Polycarpaea stylosa* Gagn. | 531.66 | 1.94 |
| 2 | *Polycarpaea arenaria* Gagn. | 763.60 | 1.94 |
| 3 | *Polycarpaea gaudichaudii* Gagnep. | 763.03 | 1.98 |
| 4 | *Polycarpaea vanphongensis* V.T. Tran, H.T. Truong, N.V. Binh | 769.32 | 1.83 |
| 5 | *Polycarpaea chungana* V.T. Tran, H.T. Truong, N.V. Binh | 441.56 | 1.86 |
| 6 | *Polycarpaea lignosa* Gagnep. | 384.63 | 1.96 |
| 7 | *Polycarpaea phuquocensis* V.T. Tran, H.T. Truong, N.V. Binh | 867.61 | 1.89 |
