# Supplementary material for: Molecular phylogeny and cryptic morphology: A combined approach to taxonomic novelties in Polycarpaea (Caryophyllaceae) from Vietnam
Source: PLoS One. 2024 Oct 16;19(10):e0301407. doi: 10.1371/journal.pone.0301407 (PMC11482727; doi:10.1371/journal.pone.0301407)
Supplement: Attachment — Submitted filename: Response to Reviwers.doc [file pone.0301407.s006.doc]

**REBUTTAL LETTER TO ACADEMIC EDITOR AND REVIEWERS**

April 26th 2024

Dear - Dr. Waqas Khan Kayani, Academic editor

- Anonymous reviewers

I am happy to thank you very much for our article, which has been evaluated and revised a minor. I would like also to thank you and the anonymous reviewers for constructive comments and suggestions, and now I would like to respond to each point raised by

**Academic editor and reviewers:**

***1. Please ensure that your manuscript meets PLOS ONE's style requirements, including those for file naming:***

- I tried to set up my manuscript to meet PLOS ONE's style requirements.

***2. In your Methods section, please provide additional information regarding the permits you obtained for the work. Please ensure you have included the full name of the authority that approved the field site access and, if no permits were required, a brief statement explaining why.***

- In line numbers 79-80, I have written in yellow: All locations are not protected in any way.

***3. Thank you for stating the following financial disclosure:***

"This research was funded by grants from the Vingroup Innovation Foundation (VINIF.2020.DA04)."

- The funders supported money for all the study and publishing but had no role in study design, data collection, and analysis, the decision to publish, or the preparation of the manuscript.

***4. Thank you for stating the following in the Acknowledgments Section of your manuscript:***

"This research was funded by grants from the Vingroup Innovation Foundation (VINIF.2020.DA04). The first author (Ph.D. Student) would like to thank Mr. Tran Kim Chung and CT Group for their kindness in granting scholarships."

- The Acknowledgments Section indicated above has not appeared in the Acknowledgments section of my manuscript.

***5. Thank you for stating the following in your Competing Interests section: "No"***

- I stated that: "The authors have declared that no competing interests exist." as detailed online and included in my cover letter.

***6. Please provide a complete Data Availability Statement in the submission form:***

- All data are in the manuscript and/or supporting information files.

***7. The data availability statement:***

- The authors decide on a data sharing plan before acceptance

***8. Figure 1 in my submission***

- The figure 1 is removed

***9. We are unable to open your Supporting Information file [Supporting Information.rar]***

- I re-upload Supporting Information file as loose format.

***10. Please review your reference list to ensure that it is complete and correct:***

- References list is completed and corrected.

***11. Reviewers' comments:***

Reviewer's Responses to Questions

**Reviewer #1:**

*• In Abstract the names of new species should be written in full form along with their author citations.*

- I have written the names of new species in complete form (red color)

*• Keywords are missing from manuscript.*

- I have consulted similar articles published on PLOS ONE and did not see keywords appear, so we did not enter keywords in the manuscript.

*• Introduction*

- The introduction is based on the latest updated literature consulted on research of the genus *Polycarpaea* at a national or regional scale. Furthermore, there are very few studies on the taxonomy of the genus *Polycarpaea*, not only in the region but also in Vietnam. There are only two authors in Vietnam, Gagnepain (1909, 1939) and Pham (1993). Therefore, it is very convenient for us to study and compare the specimens we have collected with documents describing new species and specimens deposited at the herbaria by Gagnepain (1909, 1939) and Pham (1993). In addition, this is a new species description, so we looked at closely related species for comparison. All the references to the genus *Polycarpaea* appear in the manuscript.

- For the collection methodology in introduction section: These are usually described in the introduction to new species studies.

*• In methodology section the ecology of species*

- About describing the ecological characteristics of the three new species we added in the introduction section from lines 46-57 (red color).

- In line number 73 it is mentioned that morphological characters are studied under light microscope: we have added (red color).

- There is no information mentioned regarding phenology of the species season of collection and condition of soil during plant collection: We added it at the after of the description (red color).

**Line number 207: Phenology.** The plants were flowering and fruiting between September and November.

**Line number 253: Phenology.** The plants were flowering and fruiting between August and November.

**Line number 303: Phenology.** The plants were flowering and fruiting between November and ‎March.

*• I would like to see brief discussion of the area where the new species are found*

About describing the ecological characteristics of the three new species: We added in the introduction section from lines 47-58.

**Line number 47-58:** Specimens of branches and flowering branches were collected and represented by the collection VinIF0312, VinIF0029, and VinIF0388 (Table 1). The ranges of the three collections fall into three regions. One of these is the population of collection VinIF0029 growing sparsely scattered in the grass vegetation on the backdune, which is the climatic type of semi-arid in the south center. The population of collection VinIF.0312 is growing sparsely scattered in the shrubby vegetation on the foredune, which is the climatic type of arid in the north-south. The population of collection VinIF.0388 is growing sparsely scattered in the casuarina plantation, which is the climatic type of semi-arid in Phu Quoc Island, South Vietnam. All collected specimens were dissected and studied. The analysis results show that vegetative and reproductive characteristics are similar to the abovementioned characteristics. However, there could not be keyed out to any named species from Vietnam and neighboring regions.

Fig. Grass-Vegetation of collection VinIF.0029

Fig. Shrubby-Vegetation of collection VinIF0312

Fig. Casuarina Plantation-Vegetation of collection VinIF.0388

*• Holotype of new species should be mentioned along with the illustration of new species.*
Holotype of new species mentioned along with the illustration of new species:

- Line number 227: **Fig 4.** Holotype of *Polycarpaea vanana* V.T. Tran, H.T. Truong, N.V. Binh

- Line number 272: **Fig 6.** Holotype of *Polycarpaea duongana* V.T. Tran, H.T. Truong, N.V. Binh

- Line number 314: **Fig 8.** Holotype of *Polycarpaea chungana* V.T. Tran, H.T. Truong, N.V. Binh

*• Taxonomic keys are also important in description of new species*

- We agree that the taxonomic key is essential for describing new species. However, this result was clearly shown in the Phylogeny and Discussion section, so we did not include it. Next time, we will be studying this revised genus in Vietnam and add a classification key.

**Reviewer #2:**

*• Molecular data lacs DNA extraction figures, markers amplification of PCR and sequencing. Relative efficiency of rps16 and other markers for phylogeny. Phyllograms based of molecular markers:*

**S1 Table. The quality and quantity of total extracted genomic DNA**

| **Sample No.** | **Species** | **DNA concentration (ng/µL)** | **Ratio**  **260/280** |
| --- | --- | --- | --- |
| 1 | *Polycarpaea stylosa* Gagn. | 531.66 | 1.94 |
| 2 | *Polycarpaea arenaria* Gagn. | 763.60 | 1.94 |
| 3 | *Polycarpaea gaudichaudii* Gagnep. | 763.03 | 1.98 |
| 4 | *Polycarpaea vanana* V.T. Tran, H.T. Truong, N.V. Binh | 769.32 | 1.83 |
| 5 | *Polycarpaea chungana* V.T. Tran, H.T. Truong, N.V. Binh | 441.56 | 1.86 |
| 6 | *Polycarpaea lignosa* Gagnep. | 384.63 | 1.96 |
| 7 | *Polycarpaea duongana* V.T. Tran, H.T. Truong, N.V. Binh | 867.61 | 1.89 |

M	1	2	3	4	5	6	7

1	2	3	4	5	6	7	M

1	2	3	4	5	6	7	M

**S3 Fig.** The quality of total extracted genomic DNA and PCR products. a, total extracted genomic DNA; b, ITS1-5.8S rRNA-ITS2 PCR products; c, *rps*16 intron PCR products. M, DNA ladder (Bioline: a, 1kb; b, c, 100bp).

**S4 Fig.** The quality of DNA sequencing products. a, ITS1-5.8S rRNA-ITS2 sequences; b, *rps*16 intron sequences.

This letter is to indicate my response to Reviewer's comments. I hope that my revised manuscript can satisfy your requirements, and I would welcome any comments.

Thank you very much for your help.

Sincerely yours,

On behalf the authors

Tran Van Tien

Dalat University, Vietnam

E-mail: tientv@dlu.edu.vn
